# Improvement of Glucose Tolerance by Food Factors Having Glucagon-Like Peptide-1 Releasing Activity

**DOI:** 10.3390/ijms22126623

**Published:** 2021-06-21

**Authors:** Tohru Hira, Aphichat Trakooncharoenvit, Hayate Taguchi, Hiroshi Hara

**Affiliations:** 1 Research Faculty of Agriculture, Hokkaido University, Sapporo 060-8589, Japan; 2 Graduate School of Agriculture, Hokkaido University, Sapporo 060-8589, Japan; trakooncharoenvit.aphichat.p0@elms.hokudai.ac.jp; 3 School of Agriculture, Hokkaido University, Sapporo 060-8589, Japan; hayate-taguchi@eis.hokudai.ac.jp; 4 Department of Food Science and Human Nutrition, Fuji Women’s University, Ishikari-shi 061-320, Japan; hara@fujijoshi.ac.jp

**Keywords:** GLP-1, dietary peptides, flavonoids

## Abstract

Glucagon-like peptide-1 (GLP-1) is a gastrointestinal hormone released from enteroendocrine L cells in response to meal ingestion. GLP-1 receptor agonists and GLP-1 enhancers have been clinically employed to treat diabetes owing to their glucose-dependent insulin-releasing activity. The release of GLP-1 is primarily stimulated by macronutrients such as glucose and fatty acids, which are nutritionally indispensable; however, excessive intake of sugar and fat is responsible for the development of obesity and diabetes. Therefore, GLP-1 releasing food factors, such as dietary peptides and non-nutrients, are deemed desirable for improving glucose tolerance. Human and animal studies have revealed that dietary proteins/peptides have a potent effect on stimulating GLP-1 secretion. Studies in enteroendocrine cell models have shown that dietary peptides, amino acids, and phytochemicals, such as quercetin, can directly stimulate GLP-1 secretion. In our animal experiments, these food factors improved glucose metabolism and increased GLP-1 secretion. Furthermore, some dietary peptides not only stimulated GLP-1 secretion but also reduced plasma peptidase activity, which is responsible for GLP-1 inactivation. Herein, we review the relationship between GLP-1 and food factors, especially dietary peptides and flavonoids. Accordingly, utilization of food factors with GLP-1-releasing/enhancing activity is a promising strategy for preventing and treating obesity and diabetes.

## 1. Introduction

Glucagon-like peptide-1 (GLP-1) [1] is a gastrointestinal hormone produced in enteroendocrine cells, termed L cells or GLP-1-producing cells. GLP-1 is released in response to meal ingestion (generally within 15–30 min) and enhances insulin secretion from pancreatic beta-cells, which contributes to normalizing (attenuating) the postprandial glycemic response. Due to its insulinotropic effects, GLP-1 is also called the “incretin” hormone. Another gastrointestinal hormone, glucose-dependent insulinotropic polypeptide (GIP), also functions as an incretin. GIP is released from other enteroendocrine cells, termed as K cells, in response to nutrient ingestion. GIP is produced in the proximal small intestine, and released GIP stimulates GLP-1 secretion. This evidence highlights the role of “a proximal-to-distal GIP-GLP-1 axis” in postprandial incretin responses [1].

GLP-1 has multiple actions, including promoting beta-cell growth, protecting beta-cell apoptosis, and reducing glucagon secretion from alpha-cells in the pancreas. In addition, GLP-1 demonstrates several extra-pancreatic actions, such as suppressing gastric emptying and food intake [1]. As GLP-1 and GIP are immediately degraded by a peptidase, i.e., dipeptidyl peptidase-4 (DPP-4), in the plasma and tissues, their half-life is estimated to be 1–2 min and 7 min, respectively [2,3,4].

More importantly, owing to its incretin action, GLP-1 receptor agonists (GLP-1 RAs) developed from a DPP-4 resistant GLP-1 analog (exenatide or exendin-4 identified from the saliva of a venomous lizard) are currently utilized in type-2 diabetes therapy [5]. Most GLP-1RAs need to be administered as injections (daily or weekly); however, an orally available GLP-1RA has been recently developed. Oral semaglutide is co-formulated with sodium *N*-(8-(2- hydroxybenzoyl) amino) caprylate (SNAC) and is absorbed through the stomach mucosa to exert its biological effect as a GLP-1RA [6]. Furthermore, DPP-4 inhibitors [7], such as sitagliptin and vildagliptin, are employed to treat type-2 diabetes as orally effective incretin (both GLP-1 and GIP) enhancers. GLP-1 RAs, but not DPP-4 inhibitors, reportedly promote a reduction in calorie intake and body weight due to the appetite-suppressing effect of GLP-1 receptor activation [5]. Accordingly, GLP-RAs and DPP-4 inhibitors are a well-established class of glucose-lowering agents for treating type 2 diabetes.

The efficacy of GLP-1 RAs and DPP-4 inhibitors in normalizing glucose metabolism indicates the possibility of GLP-1 and GIP deficiencies under conditions of obesity and/or glucose intolerance. Some early studies reported that GLP-1 secretory responses to oral glucose load or meal ingestion were diminished in individuals presenting with obesity, glucose intolerance, or diabetes when compared with those observed in healthy subjects [8,9].

However, results from subsequent human and animal studies were not consistent in terms of impaired GLP-1 secretion under these conditions [10,11,12,13,14,15]. In our animal studies [16,17,18,19], GLP-1 secretory responses to meal ingestion were consistently higher in diet-induced obese rats than in control rats. An enhanced GLP-1 response during the development of glucose tolerance and obesity could afford a protective effect against glucose intolerance [20]. In addition, GLP-1RAs are clinically effective in normalizing glycemia in patients with diabetes and obesity.

These findings indicate that increasing endogenous GLP-1 secretion by using orally administered drugs or food factors could be a novel and promising strategy for preventing glucose intolerance or improving glucose tolerance. Thus, in the present review, we focus on the effects of dietary factors on GLP-1 stimulation.

## 2. Dietary Factors and GLP-1

### 2.1. Macronutrients

The ingestion of a meal plays a major role in stimulating GLP-1 secretion. GLP-1-producing enteroendocrine cells (L cells) are scattered throughout the epithelium of the small and large intestines. Although GLP-1-producing cells are primarily located in the distal small intestine and the large intestine (cecum and colon), they are also present in the jejunum [21,22]. Therefore, GLP-1-producing cells in the small intestine are likely responsible for the acute postprandial GLP-1 responses induced by luminal nutrients [23]. In contrast, GLP-1-producing cells in the large intestine are possibly involved in prolonged or basal GLP-1 secretion promoted by the luminal content in the large intestine, such as short-chain fatty acids (SCFAs) produced by gastrointestinal microbiota [23].

Among macronutrients, glucose is the best-known molecule responsible for stimulating GLP-1 secretion [23], with a stimulatory mechanism similar to glucose-induced insulin secretion in pancreatic beta-cells. Glucose is taken up into GLP-1-producing cells, as well as into absorptive epithelial cells, through the sodium-dependent glucose transporter-1 (SGLT-1). In GLP-1-producing cells, glucose is metabolized to produce ATP, which induces the closure of the ATP-sensitive potassium channel (K_ATP_ channel). K_ATP_ channel closure leads to the opening of a depolarization-dependent calcium channel, resulting in intracellular calcium mobilization and subsequent GLP-1 exocytosis. Although the molecular mechanisms remain poorly understood, fructose is also a potent GLP-1 stimulator [24]. Additionally, the involvement of the sweet taste receptor (G-protein-coupled receptor (GPCR) heterodimer TAS1R2 and TAS1R3) has been proposed for sensing glucose and sweeteners [25,26,27].

Long-chain fatty acids liberated from dietary triacylglycerol potently stimulate GLP-1 secretion through GPCRs such as FFRA1 (GPR40) and FFAR4 (GPR120). In terms of the chain-length dependency, fatty acids with more than 12 chains demonstrate a GLP-1 releasing effect [28]. Previous human studies demonstrated an olive oil-rich meal induced a higher GLP-1 response than a butter-rich meal [29,30], suggesting that unsaturated fatty acids are more effective stimulators of GLP-1 secretion than saturated fatty acids. However, structure-activity relationships regarding the degree of unsaturation and positions of the double bond remain unclear. 2-Oleoil glycerol (2-OG) is a specific monoacylglycerol known to stimulate GLP-1 secretion through GPR119, which is also activated by the fatty acid derivative oleoylethanolamide (OEA) [31,32].

Protein-, peptide- and amino acid-induced GLP-1 secretions are separately described below.

### 2.2. Micronutrients

The direct effects of vitamins and minerals on gastrointestinal hormone secretion, including GLP-1 secretion, remain unknown. One possible mineral that stimulates GLP-1 secretion is calcium, as the extracellular calcium-sensing receptor (CaSR) is expressed and functions in GLP-1-producing cells as a sensor for specific amino acids and peptides [33,34]. However, whether dietary or luminal calcium alone induces GLP-1 secretion needs to be clarified.

### 2.3. Other Food Factors

In the large intestine, fermentable dietary fibers are a major source of SCFAs, such as acetate, propionate, and butyrate. The stimulatory effects of chronic dietary fiber ingestion on GLP-1 secretion have been reported in various animal and human studies, as summarized in a previous review [35]. Previous reports [36,37] have demonstrated that acetate and propionate stimulate GLP-1 secretion through the free fatty acid receptor 2 (FFAR2, also called GPR43) and free fatty acid receptor 3 (FFAR3, also called GPR41). Another study revealed that SCFAs are intracellularly metabolized to produce energy, subsequently triggering GLP-1 secretion [38].

Continuous feeding with resistant maltodextrin (RMD) increased plasma GLP-1 concentrations, accompanied by increased SCFA production in normal rats and high fat/high sucrose diet-fed rats [17,39]. In a murine enteroendocrine GLP-1-producing cell line (GLUTag cells), RMD exposure for 60 min increased GLP-1 release into the supernatant, and a direct infusion of RMD into the small intestine of anesthetized rats acutely increased portal plasma GLP-1 concentrations [39]. As these experimental models do not include gut microbiota, the results suggest a direct effect of RMD molecules on GLP-1 secretion in GLP-1-producing cells.

D-allulose, a non-metabolizable sugar, exerts potent GLP-1 release in rat and mouse models [40,41]. Oral administration of D-allulose restricted overeating and hyperglycemia by increasing GLP-1 secretion in mice. Interestingly, D-allulose induced GLP-1 secretion but not affected GIP secretion. Most macronutrients stimulate both GLP-1 and GIP secretion, and GIP promotes adiposity [4], an undesirable action during obesity. The GLP-1-specific action of D-allulose is an attractive property for the prevention and amelioration of obesity and diabetes. The effects of RMD and D-allulose on GLP-1 secretion in humans and the mechanism underlying RMD and D-allulose-induced GLP-1 secretion need to be comprehensively investigated in future studies.

The effects of phytochemicals on GLP-1 are separately described below.

## 3. Dietary Proteins/Peptides/Amino Acids and GLP-1

### 3.1. Proteins

Recently, dietary proteins and peptides have been recognized as potent GLP-1 secretagogues [42], as reported in several animal and human studies. Meals composed of higher amounts of protein stimulate GLP-1 secretion substantially [43,44,45] and are associated with reduced glycemia and/or enhanced satiety. However, this may not always be observed, as it is difficult to control the experimental design under iso-caloric conditions. In order to adjust for identical energy in test meals (isocaloric condition), on increasing the protein proportion, the carbohydrate and/or fat proportion needs to be decreased, which are also potent GLP-1 stimulators. Furthermore, it is difficult to ingest adequate amounts (e.g., 75 g) of pure protein when compared with 75 g glucose (typical dose used in human glucose tolerance tests), as most dietary proteins are insoluble in water and poorly palatable. Therefore, human studies directly comparing the effects of pure macronutrients are limited [46].

On comparing the effects of isocaloric (8 kcal/kg body weight) glucose, fat (olive oil), and protein (whey protein and egg protein mixture) on GLP-1 secretion in non-diabetic subjects, fat reportedly induced the highest increment in GLP-1 secretion [46]. As described above, whey protein is predominantly used in human studies and exerts a GLP-1 stimulating action, followed by insulinotropic and glucose-lowering effects. Accordingly, whey protein is now employed as a positive control to stimulate GLP-1 secretion.

Some studies have demonstrated the effects of intact proteins on GLP-1 secretion in enteroendocrine cell models (STC-1 and pGIP/Neo STC-1 cells). On examining various intact proteins, casein, codfish, egg, and wheat revealed potent effects on GLP-1 secretion from STC-1 cells [47]. Among whey protein components (α-lactalbumin and β-lactoglobulin), α-lactalbumin is potently stimulated [48], and among casein subunits (α-, β, κ-casein), α- and β-casein stimulated GLP-1 secretion from pGIP/Neo STC-1 cells [49]. In GLUTag cells, the micellar casein concentrate, but not sodium caseinate mixed with whey protein isolate, induced GLP-1 secretion [50].

As cell lines are incapable of digesting these proteins, these findings indicate that specific polypeptide structures, not only peptides and free amino acids, are involved in the stimulatory effect on GLP-1 secretion.

### 3.2. Peptides

As dietary proteins are digested in the stomach and intestinal lumen, digestive products such as peptides and amino acids stimulate the GLP-1 producing cells in the intestine. Some dietary protein hydrolysates reportedly stimulate GLP-1 secretion in humans and animal models.

Acute intraduodenal infusion of whey protein hydrolysates (3 kcal/min for 60 min, total 180 kcal = 45 g) increased plasma GLP-1 levels in healthy men [51], as well as in both lean and obese subjects [52]. Chronic ingestion of fish (blue whiting) protein hydrolysate (1.4 g/day), added to a meal for 90 days [53], increased plasma GLP-1 levels in marginally overweight subjects, whereas cod protein hydrolysate ingestion (~40 mg/kg body weight/day) for 1 week did not affect GLP-1 levels in older adults [54].

Mechanisms underlying increased plasma GLP-1 levels in response to acute and chronic treatment with these factors are not necessarily common, as the former is a single stimulus–secretion coupling, but the latter includes adaptation to repeated stimulation. The adaptive mechanisms of dietary conditions in enteroendocrine cells remain largely elusive when compared with stimulus–secretion coupling mechanisms.

Oral administration of corn zein hydrolysate [55] and rice protein hydrolysate [56] stimulated GLP-1 secretion, which was accompanied by increased insulin secretion and decreased glycemia in rats, including diabetic Goto-Kakizaki rats [55]. Overall, dietary protein hydrolysates commonly stimulate GLP-1 secretion, although the relative potency varies among ingredients. Such variations are possibly caused by experimental conditions, including protein sources, enzymes used, duration, and combinations of digestive enzymes (such as pepsin, trypsin, chymotrypsin, pancreatin).

Owing to recent advances in peptidomic analysis using liquid chromatography with tandem mass spectrometry (LC-MS/MS), amino acid sequences of fragments in protein hydrolysates have been comprehensively decoded. Although limited in number and identifying very short peptides are technically challenging [57,58], peptide fragments with GLP-1 releasing activity have been identified, as shown in Table 1. Length of peptides vary from di- to undeca-peptide, and they do not seem to have any common structures or features.

Selected tetrapeptides were identified to induce GLP-1 release from the human enteroendocrine cell line NCI-H716 [59]. A recent study demonstrated that a hydrophobic, positively charged peptide from egg white protein possesses potent GLP-1 releasing activity [60].

**Figure 1:**
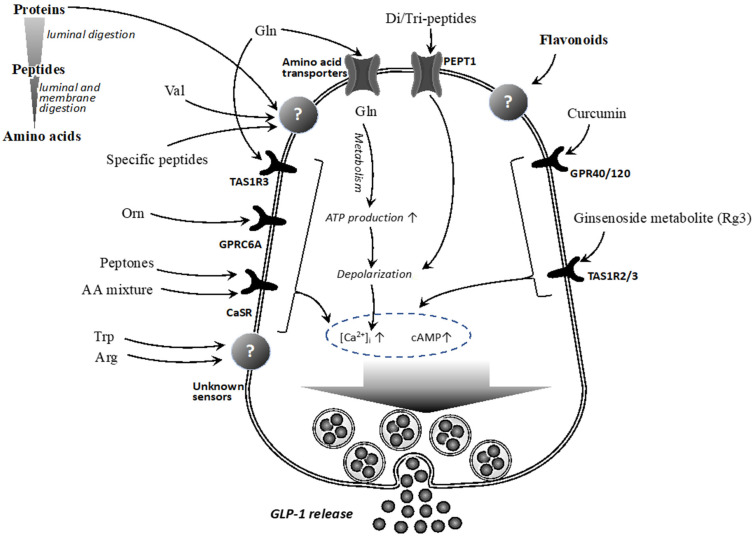
Sensing mechanisms of dietary proteins/peptides/amino acids and flavonoids in GLP-1-producing cells. Abbreviations; Gln, glutamine; Val, valine; Orn, ornithine; AA mixture, amino acids mixture; Trp, tryptophane; Arg, arginine; [Ca^2+^]_i_, intracellular calcium concentration; cAMP, cyclic adenosine mono-phosphate.

However, GLP-1-releasing peptides possibly possess specific features, as only limited but not all peptide fragments have the activity. Molecular mechanisms, especially the sensing mechanisms of dietary peptides in GLP-1-producing cells, have been partially clarified, as summarized in Figure 1.

Peptide transporter-1 (PEPT1) is reportedly involved in GLP-1 secretion induced by glycylsarcosine (the non-hydrolyzable dipeptide PEP1 substrate) in L cells isolated from GLU-Venus transgenic mice, based on the cell-specific expression of the fluorescent protein Venus [33]. The involvement of CaSR has also been shown in peptone (an enzymatic meat hydrolysate)-induced GLP-1 secretion [33,34]; however, specific peptides have not been identified.

Numerous peptides are liberated from dietary proteins during luminal digestion in the stomach and small intestine. The degree of hydrolysis differs depending on the substrate, duration, amount of luminal enzymes, and intestinal region (duodenum, jejunum, ileum). The amount of luminal peptidases derived from the exocrine pancreas (trypsin, chymotrypsin, elastase, carboxypeptidases) is regulated by cholecystokinin (CCK), which is released from enteroendocrine I cells mainly located in the proximal small intestine, in response to nutrient ingestion, including fat and protein [61]. Therefore, it is challenging to mimic or estimate the components of luminal peptides in distinct intestinal regions.

Some active peptides might be released during the early period of luminal digestion in the proximal small intestine; however, they might be further digested and lose their activity before they reach the distal small intestine where GLP-1-producing cells are abundantly present. On the other hand, some small active peptides might be liberated after sufficient digestion in the small intestine, but they are immediately absorbed into epithelial cells before acting on GLP-1-producing cells. Despite these limitations, INFOGEST [62], a static in vitro simulation of gastrointestinal food digestion, helps identify and estimate peptide fragments after luminal digestion [60].

Although it has been postulated that luminal peptides act on CaSR located on the apical side of enteroendocrine cells to trigger GLP-1 secretion, a recent study using the isolated perfused proximal rat small intestine indicated that peptone-induced GLP-1 secretion depends on intestinal absorption and activation of basolaterally located CaSR [63]. Further investigations are needed to identify the active peptides and receptors involved in dietary protein hydrolysate-induced GLP-1 secretion.

In addition to stimulating GLP-1 secretion, dietary peptides are considered to enhance the survival of biologically active GLP-1 by reducing (inhibiting) DPP-4 activity in plasma. Sitagliptin, a non-peptidomimetic molecule, is an orally effective DPP-4 inhibitor that is widely employed as an incretin enhancer for the treatment of diabetes [7]. Although its potency is substantially lower than that of sitagliptin (IC50, 0.2 µM), some small peptides such as diprotin A (Ile-Pro-Ile) have been known to inhibit DPP-4 activity (IC50, 120 µM) [64]. Many small peptides derived from various dietary proteins demonstrate DPP-4 inhibitory activity, as observed in in vitro studies [65,66]. However, it remains unclear whether these peptides play a role in inhibiting plasma DPP-4 activity, followed by an increase in plasma GLP-1 levels in vivo.

The direct administration of corn zein hydrolysate [67] and rice protein hydrolysate [56] in anesthetized rats reduced plasma DPP-4 activity in the ileal mesenteric vein plasma. Furthermore, oral administration of whey protein stimulated GLP-1 secretion and afforded protection against GLP-1 degradation by DPP-4 in awake rats in experiments comparing GLP-1 responses to whey protein and dextrin with or without DPP-4 conditions [68]. This hypothesis is supported by similar experimental results observed in a previous mouse study [69], but it has not been evident in humans [70]. This could be attributed to the technical limitations of human studies, with only peripheral blood samples are available to assess DPP-4 activity. It can be postulated that the DPP-4 inhibitory and GLP-1-enhancing effects occur locally in the mesenteric blood. The GLP-1-releasing and GLP-1-enhancing effect of whey protein could be involved in its insulinotropic effect [71].

### 3.3. Amino Acids

Not all amino acids stimulate GLP-1 secretion. Glutamine (Gln) was the first identified amino acid demonstrating potent GLP-1 releasing activity in murine GLUTag cells [73]. Subsequent studies revealed the involvement of elevated cytosolic calcium and cAMP in Gln-induced GLP-1 secretion in L cells [74]. Based on these studies, the involvement of a specific Gln receptor was suggested. One taste receptor subunit, TAS1R3, has been reported to be involved in Gln-induced GLP-1 secretion in GLUTag cells [75]. In contrast, another research group has suggested that Gln was transported into L cells and that the intracellular metabolism mediated by glutamate dehydrogenase (GDH) plays a role in Gln-induced GLP-1 secretion [76].

Ornithine (Orn) is a non-protein amino acid that potently stimulates GLP-1 secretion in GLUTag cells [77] and mice [78]. The involvement of a GPCR (GPRC6A) was demonstrated in GLUTag cells [77]; however, a study using GPRC6A knockout mice did not fully support this mechanism [78].

A recent study using the perfused proximal rat small intestine demonstrated that valine (Val) and Gln potently stimulated GLP-1 secretion only from the luminal side, but not from the vascular side, while arginine (Arg) and tryptophan (Trp) did only from the vascular side [79]. Accordingly, they concluded that amino acid-induced GLP-1 secretion involves both apical and basolateral (post-absorptive) sensing mechanisms. The apical sensing mechanism remains unknown, but the involvement of CaSR has been implicated in basolateral sensing mechanisms.

Human studies have revealed the stimulatory effect of orally administered Gln (30 g) on GLP-1 secretion in lean individuals, as well as in patients with obesity and type-2 diabetes [80], accompanied by a reduction in glycemia [81]. A clinical trial was conducted using a relatively lower dose of Gln (6 g) encapsulated with an enteric coating designed to promote capsule release 20 min after exposure to an alkaline environment [82]. Although a small increment of plasma GLP-1 was observed, it failed to exert beneficial metabolic effects on glucose tolerance and satiety. These results indicate that a 15–30 g dose of Gln is required to exert beneficial metabolic effects as a GLP-1 releaser in humans.

Considering the process of luminal digestion of dietary proteins, all factors derived from dietary proteins, including intact proteins, denatured proteins, partially digested peptides, oligopeptides, and free amino acids, can stimulate GLP-1 secretion depending on their structures and specific or unknown cellular mechanisms. Therefore, understanding protein/peptide/amino acid-induced GLP-1 secretion remains an attractive research target from a physiological perspective, as well as for the prevention and treatment of obesity and diabetes.

## 4. Phytochemicals and GLP-1

### 4.1. Anti-Diabetic Effects of Phytochemicals, and Related Mechanisms

Phytochemicals are naturally occurring compounds in plants that include a large number of chemical species. Extensive research in humans who consume phytochemical-rich foods and beverages has revealed a wide range of potential health benefits against diabetes and related diseases; however, sufficient data to recommend specific phytochemical intake are lacking [83,84,85,86]. Among the wide varieties of phytochemicals, flavonoids are the largest and most evaluated group [87]. Flavonoids are a class of low-molecular-weight phenolic compounds widely distributed in plants [88].

Notably, the anti-diabetic effects of flavonoids are partially attributed to their antioxidative properties and ability to modulate cellular signaling. Dietary genistein improved glucose tolerance and blood insulin levels in streptozotocin-induced diabetic mice, accompanied by improved islet beta-cell proliferation, survival, and mass. The beneficial effect on pancreatic beta-cells is mediated via activation of the cAMP/PKA-dependent ERK1/2 signaling pathway [89]. Both in vitro (INS-1 and MIN6 cell lines) and ex vivo (mouse pancreatic islets) models revealed that genistein directly exerted an insulinotropic effect through the cAMP/PKA signaling pathway in pancreatic beta-cells, independent of the estrogen receptor [90]. These beneficial effects of genistein have been demonstrated in postmenopausal women with type 2 diabetes mellitus [91]. However, the underlying mechanism has not been confirmed in human studies.

In a type 2 diabetic mouse model, quercetin ameliorated hyperglycemia and oxidative stress by lowering free radical-induced toxicity [92]. A co-formulation composed of quercetin and sitagliptin restored pancreatic islets and beta-cell function in diabetic rats [93]. (–)-Epicatechin reportedly exerts a beneficial effect on insulin sensitivity by downregulating oxidant production pathways that are known to inhibit insulin signaling [94]. Treatment with rutin reduced plasma glucose levels and attenuated oxidative stress and neuroinflammation via activation of the nuclear factor erythroid 2-related factor 2 (Nrf-2) pathway in the dorsal root ganglia of streptozotocin-induced diabetic rats [95]. Furthermore, several independent studies have demonstrated pancreatic beta-cell survival and/or pancreatic insulin release induced by phytochemicals such as fisetin [96], proanthocyanidin [97], and naringenin [98] in oxidative stress models.

Some flavonoids reportedly inhibit carbohydrate digestion, absorption, and metabolism. Tiliroside inhibited pancreatic α-amylase in a dose-dependent manner and suppressed glucose uptake mediated by both SGLT1 and GLUT2 in the gastrointestinal tract [99]. Quercetin, epigallocatechin gallate (EGCG), and epicatechin gallate (ECG) inhibited the facilitative glucose transporter, GLUT1, by interacting at its exofacial sugar-binding site [100]. Naringenin [101], tangeretin [102], tricin [103], and quercetin [104] promoted glucose absorption in insulin-sensitive tissues via the translocation of GLUT4. Fisetin is another flavonoid that improved glucose homeostasis in streptozotocin-induced diabetic rats by inhibiting hepatic gluconeogenic enzymes (phosphoenolpyruvate carboxykinase and glucose-6-phosphatase) [105]. In addition, rutin [106], kaempferol [107], and hesperidin [108] improved glucose tolerance by modifying carbohydrate metabolic enzymes.

### 4.2. Phytochemicals and GLP-1

As listed in Table 2, various flavonoids reportedly stimulate GLP-1 secretion in intestinal cell models and intestinal tissues and elevate plasma GLP-1 concentrations in animal models. As expected, most animal studies have shown improvements in glucose tolerance, along with increased plasma GLP-1 concentrations.

Using enteroendocrine cell models (murine GLUTag and human NCI-H716), stimulatory effects of curcumin [109], delphinidin 3-rutinoside [110], ginsenoside metabolite, Rg3 [111], hispidulin [112], and isoquercitrin [113] have been demonstrated. Although not well recognized, human Caco2 cells were employed to demonstrate GLP-1 secretion induced by EGCG [114]. Additionally, this study revealed the GLP-1 releasing activity of EGCG in a mouse intestinal tissue segment model.

Interestingly, these compounds exert a stimulatory effect at relatively lower concentrations (µM or µg/mL) than dietary peptides and amino acids that require mM or mg/mL concentration ranges, as described above (Table 1).

However, the molecular mechanisms (Figure 1) by which these flavonoids activate cells to release GLP-1 have not been comprehensively elucidated. To date, the involvement of free fatty acid receptors GPR40 and/or GPR120 in curcumin-induced GLP-1 secretion [116], and the sweet taste receptor in ginsenoside metabolite, Rg3-induced GLP-1 secretion [109], have been proposed.

In animal studies, GLP-1 secretion can be induced by acute (single) administration of curcumin [116], as well as the ginsenoside metabolite Rg3 [111]. Increment of plasma GLP-1 levels following chronic administration (oral or intraperitoneal) of apigenin [115], genistein combined with metformin [117], hispidulin [112], isoquercitrin [113], luteolin [118], myricetin [119], grape seed proanthocyanidins [120], procyanidin [121], and resveratrol [122] have been reported.

Some compounds (isoquercitrin and myricetin) reportedly possess inhibitory activity against the DPP-4 enzyme, which is considered to contribute to the promotive effect on GLP-1 levels [113,119]. Flavonoids mediate various protective effects against obesity, diabetes, and other metabolic diseases by targeting various organs, tissues, and cells, as described previously. Accordingly, the promotive effects of flavonoids on GLP-1 may, in part, mediate their health beneficial effects.

Most studies used purified flavonoids or flavonoid-enriched extracts. It is unlikely that humans have a sufficient amount of flavonoids from a single meal containing “flavonoid-rich foods” for achieving the effective concentrations (µM or µg/mL range) in the intestinal lumen for stimulating GLP-1secretion. One previous human study demonstrated that the consumption of applesauce containing onion powder (∼100 mg of quercetin aglycone equivalents) resulted in a maximum plasma quercetin concentration at 0.9 µM [123]. Thus, in order to promote flavonoid-induced GLP-1 secretion, we would currently need to use purified- or concentrated flavonoids. However, continuous ingestion of flavonoid-rich foods may accumulate active flavonoids in the intestinal lumen or the blood circulation if the intestinal degradation of flavonoids was suppressed and the bioavailability of flavonoids was enhanced by coingestion of flavonoids with other food factors such as fermentable dietary fibers [124,125].

## 5. Concluding Remarks

Lowering plasma glucose levels is considered a major target for treating or preventing glucose impairment, except when the possibility of hypoglycemia exists. Pharmaceutical approaches are potently effective but limited only to “patients” with a medical diagnosis. Nutraceutical approaches are not always efficacious but can be employed in subjects with pre-symptomatic status. Food factors targeting the liver, muscle, adipose tissues, and blood vessels need to be absorbed through the intestine and delivered to target cells via blood circulation.

Safety and bioavailability must be considered when such food factors are utilized in humans. However, when intestinal epithelial cells, including enteroendocrine cells, are targeted, especially when food factors act on the apical side, these factors do not need to cross the intestinal epithelium.

As reviewed above, GLP-1 secretion is stimulated by various food factors, indicating that, when needed, GLP-1 secretion can be increased by these food factors.

Thus, the utilization of low-digestible and/or low-absorbable materials, such as flavonoids and slowly digestible peptides, is a promising and safe strategy for the treatment and prevention of metabolic diseases, including obesity and glucose intolerance, by increasing GLP-1 secretion.

## Figures and Tables

**Table 1:** Sequences and effective concentrations of dietary protein-based peptides and synthetic peptides that have GLP-1 releasing activity in various cellular models.

Peptide Sequence	Models	Concentration Used (mM)	Source	Reference
**TKAVEH**	STC-1	0.1	bovine hemoglobin	Caron, J. Food Res Int 2016 [72]
**ANVST**	STC-1	1	bovine hemoglobin	Caron, J. Food Res Int 2016 [72]
**KAAVT**	STC-1	1	bovine hemoglobin	Caron, J. Food Res Int 2016 [72]
**YGAE**	STC-1	1	bovine hemoglobin	Caron, J. Food Res Int 2016 [72]
**GPVRGPFPIIV**	GLUTag	5	β-casein	Komatsu, Y. Food Chem 2019 [50]
**PFL**	STC-1	2	egg white protein	Santos-Hernández, M. Food Chem 2020 [60]
**RVASMASEKM**	STC-1	2	egg white protein	Santos-Hernández, M. Food Chem 2020 [60]
**LKPT**	STC-1	1	tilapia byproduct	Theysgeur, S. Molecules 2020 [58]
**GGGG**	NCl-H716	20	synthetic	Le Nevé, B. Regul Pept 2011 [59]
**AAAA**	NCl-H716	10	synthetic	Le Nevé, B. Regul Pept 2011 [59]
**GWGG**	NCl-H716	10	synthetic	Le Nevé, B. Regul Pept 2011 [59]
**Glycylsarcosine**	Murine colon primary culture	10	synthetic	Diakogiannaki, E. Diabetologia 2013 [33]
**LGG**	Murine colon primary culture	10	synthetic	Diakogiannaki, E. Diabetologia 2013 [33]
**GF**	Murine colon primary culture	10	synthetic	Diakogiannaki, E. Diabetologia 2013 [33]

Amino acid sequences are presented by one letter abbreviations, except for glycylsarcosine.

**Table 2:** Flavonoids reported to elevate GLP-1 levels.

Compound	Models	Treatment	Effect	Reference
**Apigenin**	High fat-high fructose diet-fed rats	1.5 mg/kg BW, intraperitoneal, 30 days	Plasma GLP-1↑	Kalivarathan, J. J Func Foods 2020 [115]
**Curcumin**	GLUTag cells	10–50 µM, 2 h	GLP-1 secretion↑	Takikawa, M. Biochem Biophys Res Commun 2013 [109]
**Curcumin**	Rats	1.5 mg/kg BW, oral	Plasma GLP-1↑ Glucose tolerance↑	Kato, M. Mol Nutr Food Res 2017 [116]
**Delphinidin 3-rutinoside**	GLUTag Cells	10–100 µM, 2 h	GLP-1 secretion↑	Kato, M. PLoS One 2015 [110]
**Epigallocatechin-3-gallate**	Caco-2 cells Murine ileal tissue	300 µM, 2 h 1 mM, 45 min	GLP-1 secretion↑ GLP-1 secretion↑	Song, WY. J Clin Biochem Nutr 2015 [114]
**Genistein (combined with metformin)**	Alloxan-induced diabetic rats	20 mg/kg BW/day, intraperitoneal, 30 days	Serum and intestinal GLP-1↑ Glucose tolerance↑	Rehman, K. Biomed Pharmacother 2019 [117]
**Ginsenoside metabolite, Rg3**	NCI-H716 cells db/db mice	1–25 µM, 1 h 0.5 mg/kg BW, oral	GLP-1 secretion↑ Plasma GLP-1↑ Glucose tolerance↑	Kim, KS. Sci Rep. 2015 [111]
**Hispidulin**	GLUTag cells Mouse ileum crypts STZ-treated mice	1–50 µM, 1 h 1–50 µM, 1 h 20 mg/kg BW/day, oral, 6 weeks	GLP-1 secretion↑ GLP-1 secretion↑ Plasma GLP-1↑ Glucose tolerance↑	Wang, Y. Mol Nutr Food Res 2020 [112]
**Isoquercitrin**	NCI-H716 cells HFD-fed, STZ-treated mice	10–100 µM, 90 min 20–80 mg/kgBW/day, oral, 8 weeks	GLP-1 secretion↑ Plasma GLP-1↑ Plasma DPP-4↓ Plasma Glucose↓	Zang, L. RSC Adv 2018 [113]
**Luteolin**	HFD-fed mice	Supplemented in diet, 0.005%, 16 weeks	Plasma GLP-1↑ Glucose tolerance↑	Kwon, EY. Nutrients 2018 [118]
**Myricetin**	HFD-fed, STZ-treated rats	20 mg/kg BW/day, oral, 4 weeks	Plasma GLP-1↑ Plasma and tissue DPP-4↓	Lalitha, N. PLoS One 2020 [119]
**Proanthocyanidin Gallic acid**	Rat ileal segment	0.2 mg/mL, 1 h 31 µg/mL, 1 h	GLP-1 secretion↑ GLP-1 secretion↑	Casanova-Martí, À. Food Nutr Res 2017 [120]
**Procyanidin**	Cafeteria diet-fed rats	25 mg/kg BW/day, oral, 12 weeks	Intestinal GLP-1↑	González-Abuín, N. J Agric Food Chem 2014 [121]
**Resveratrol**	HFD-fed mice	Supplemented in diet, 60 mg/kg BW/day, 5 weeks	Plasma GLP-1↑ Glucose tolerance↑	Dao, TM. PLoS One 2011 [122]

Abbreviations: BW, body weight; DPP-4, dipeptidyl peptidase-4; GLP-1, glucagon-like peptide-1; HFD, high-fat diet; STZ, streptozotocin.
